# Can Spectral CT Imaging Improve the Differentiation between Malignant and Benign Solitary Pulmonary Nodules?

**DOI:** 10.1371/journal.pone.0147537

**Published:** 2016-02-03

**Authors:** Ying Zhang, Jiejun Cheng, Xiaolan Hua, Mingji Yu, Chengdong Xu, Feng Zhang, Jianrong Xu, Huawei Wu

**Affiliations:** Department of Radiology, Renji Hospital, School of Medicine, Shanghai Jiaotong University, Shanghai, China; University of Munich, GERMANY

## Abstract

**Purpose:**

To quantitatively assess the value of dual-energy CT (DECT) in differentiating malignancy and benignity of solitary pulmonary nodules.

**Materials and Methods:**

Sixty-three patients with solitary pulmonary nodules detected by CT plain scan underwent contrast enhanced CT scans in arterial phase (AP) and venous phase (VP) with spectral imaging mode for tumor type differentiation. The Gemstone Spectral Imaging (GSI) viewer was used for image display and data analysis. Region of interest was placed on the relatively homogeneous area of the nodule to measure iodine concentration (IC) on iodine-based material decomposition images and CT numbers on monochromatic image sets to generate spectral HU curve. Normalized IC (NIC), slope of the spectral HU curve (λ_HU_) and net CT number enhancement on 70keV images were calculated. The two-sample *t*-test was used to compare quantitative parameters. Receiver operating characteristic curves were generated to calculate sensitivity and specificity.

**Results:**

There were 63 nodules, with 37 malignant nodules (59%) and 26 benign nodules (41%). NIC, λ_HU_ and net CT number enhancement on 70keV images for malignant nodules were all greater than those of benign nodules. NIC and λ_HU_ had intermediate to high performances to differentiate malignant nodules from benign ones with the areas under curve of 0.89 and 0.86 respectively in AP, 0.96 and 0.89 respectively in VP. Using 0.30 as a threshold value for NIC in VP, one could obtain sensitivity of 93.8% and specificity of 85.7% for differentiating malignant from benign solitary pulmonary nodules. These values were statistically higher than the corresponding values of 74.2% and 53.8% obtained with the conventional CT number enhancement.

**Conclusions:**

DECT imaging with GSI mode provides more promising value in quantitative way for distinguishing malignant nodules from benign ones than CT enhancement numbers.

## Introduction

Evaluation of the solitary pulmonary nodules (SPNs) remains a substantial and costly challenge in modern medicine [1–5]. For the patients suspected with malignancy, several imaging modalities could be used for diagnosis, such as CT, angiography, positron emission tomography (PET), magnetic resonance imaging (MRI) or even Doppler ultrasonography [5]. However, a standard contrast-enhanced CT scan is often the first examination [6]. Diagnostic evaluation of SPN using conventional CT consists of two major parts: morphologic features (such as size, margins, contour, internal characteristics [7]) and degree of enhancement. Measuring the enhancement degree of SPN which is directly related to the vascularity and distribution of the intravascular and extracellular spaces, has proven to be helpful in distinguishing malignant nodules from benign ones in dynamic contrast-enhanced CT [8–11], due to the distinct differences in the vascularity and vasculature of benign and malignant nodules [12–14]. The disorder and variety of the arrangement of vessels along with large intravascular and interstitial spaces in lung cancer would contribute to rich and rapid peak enhancement and retention of contrast medium [11, 12]. Most of the inflammatory pulmonary lesions also enhance strongly because of the vascular supply from the bronchial arteries [11], and washout might be seen after peak enhancement was reached, since contrast medium would flow through relatively straight vessels with a normal configuration and be accelerated by active lymphatic flow [11]. While some uninflammatory benign nodules have slight enhancement due to the lack of vascularity.

However, there is considerable overlap between benign and malignant SPNs in terms of enhancement patterns in conventional CT [5,15]. A prospective and multicenter study including 356 pulmonary nodules (171 malignant nodules and 185 benign nodules) has shown that, using enhancement degree as a diagnostic parameter of discerning benign and malignant SPNs, the resulting specificity was only 58%, although the sensitivity to be as high as 98% [5]. Furthermore, contrast-enhanced dynamic CT has the disadvantage of an increased radiation dose to patients [16].

Dual Energy gemstone spectral imaging (GSI), however, enables the generation of material decomposition images that allows one to measure iodine component of SPNs on iodine-enhanced images, and this is considered to be comparable to the real value (net enhancement) of the extent of enhancement [6,16,17]. Moreover, the possibility of differentiating single energy levels in a polychromatic examination allows acquisition of different monochromatic image sets, which provides information about the attenuation changes in different materials as a function of x-ray photon energy [17,18].

The purpose of our study was to determine whether DECT with GSI reveals significant differences between malignant and benign SPNs.

## Materials and Methods

### Patients

This retrospective research was approved by Renji hospital institutional review board, and written informed consent was obtained from each patient. From December 2012 to April 2015, a total of 365 patients underwent routine dual-phase contrast-enhanced scans with DECT, and SPNs were detected in 97 patients. The 63 patients (37 men, 26 women; age range, 30–87 years; median age, 55 years) who had following resection or biopsy of SPN were finally included in our study.

### CT Examinations

All patients underwent CT examinations (non-enhanced and dual-phasic contrast-enhanced scans) on a Discovery CT750HD scanner (GE Healthcare, WI). The non-enhanced scanning was performed first with the conventional helical mode at a tube voltage of 120kVp; tube current, 180mA; helical pitch, 1.375. A 80–100ml (1.35 ml/kg of body weight) nonionic iodinated contrast material (Iopamidol, 370 mg/ml; Shanghai Bracco Sine Phamaceutical Co. Ltd., China) was administrated at a rate of 4.0ml/s by using a power injector, and the dual-phasic enhancement scan delay times were 35 seconds at AP and 90 seconds at VP after the beginning of contrast medium injection. The contrast-enhanced scans were carried out with the dual energy spectral CT mode with fast tube voltage switching between 80kVp and 140kVp on adjacent views during a single rotation. The other DECT parameters were the following: tube rotation time, 0.6 seconds; tube current, 600mA; helical pitch, 1.375; field of view, 500mm; collimation, 40mm; and slice thickness and interval for axial images, 5mm/5mm. Two types of images were obtained from the reconstruction of DECT imaging automatically with GSI viewer software (GE Healthcare) for each patient: the iodine-based and water-based material decomposition images and a set of monochromatic images at energies ranging from 40 to 140keV.

### Data Analysis

Two radiologists (H.W.W. and J.J.C. with 14 and 15 years of experience in chest CT, respectively) blinded to patient information and pathologic diagnosis interpreted the images and measured the quantitative parameters in a workstation (ADW4.4; GE Healthcare) independent of each other, and their disagreement on measurement was resolved by consensus.

Because different series could not be loaded simultaneously, three ROIs (the relative homogeneous area) were used to measure the nonenhanced images, AP and VP images after enhancement. The size, shape, and position of ROIs were the same by using the copy-and-paste function. The areas of the ROIs were as follows: the malignant nodules with average of 29.82±21.04mm^2^, median of 26.17mm^2^, range from 4.21 to 114.52mm^2^; and the benign nodules with average of 35.76±21.88mm^2^, median of 26.83mm^2^, range from 3.59 to 97.20mm^2^. The CT number, iodine concentration (IC) and water concentration (WC) of lesions were measured from the monochromatic 70keV images, iodine- and water- based material decomposition images, respectively after enhancement in AP and VP. To reflect the enhancement degree of SPNs, the CT numbers of net enhancement on 70keV images were calculated as the enhanced CT numbers minus the nonenhanced CT numbers which was acquired at 120kVp tube voltage; the enhanced CT numbers in 70keV monochromatic energy was selected for two reasons: firstly, 120kVp scanning in conventional polychromatic images has an average energy of about 70keV in GSI mode [19], and secondly, different from the two-tube, two detector system, the single source spectral CT mode does not produce the mixed (weighted) CT images that mimic 120kVp, instead, it produces a set of monochromatic images with energies range from 40 to 140keV.

To minimize variations caused by the patient’s circulation status and scanning times, the IC values of lung lesion was normalized to that of thoracic aorta in the same slice to derive the normalized iodine concentration (NIC): IC_lesion_/IC_aorta_. For the CT number measurement, the GSI software also automatically propagated the ROIs to all the monochromatic image sets with energies from 40 to 140keV to generate the spectral attenuation curves. The slope of such curve (λ_HU_) was calculated as the CT attenuation difference at two energy levels (40 and 100keV) divided by the energy difference (60keV) from the spectral HU curve, according to the formula: λ_HU_ = |CT_40-keV_-CT_100 keV_|/60. As in general the curve between 100 and 140keV was almost flat, we chose the range of 40 and 100keV of which the slope was larger to improve the sensitivity of describing different slopes of spectral attenuation curves than the whole range.

### Statistical Analysis

Finally, four types of data were obtained as follows: the CT numbers of net enhancement in 70keV monochromatic images, NIC, WC and λ_HU_. The results were expressed as mean ± standard deviation. Commercial statistical analysis packages (version 22.0.0 SPSS, IBM) were used to analyze the measurements. The two-sample *t*-test was used to statistically compare these parameters between cancerous and benign nodules, and paired-sample *t*-test was used to statistically compare these parameters between AP and VP for malignancy and benignity, all with *P* value <0.05 considered as statistically significant.

Receiver operating characteristic (ROC) curves were generated to establish the optimal threshold values to distinguish lung cancers from benign nodules and calculated the sensitivity and specificity. The diagnostic capability was determined by calculating the area under the ROC curve (AUC).

For the calculation of diagnostic accuracy of the degree of CT enhancement for distinguishing malignant and benign nodules, a 20HU threshold was used. We selected 20HU as a cutoff value on the basis of results of previous studies [8–10, 16]. For the other parameters, the best sensitivity and specificity were achieved by using the optimal thresholds.

## Results

### Radiation Dose

The volumetric CT dose index (CTDIvol) for each phase with DECT acquisition was a fixed value of 12.72 mGy with a fixed scan protocol because automatic exposure control was not available for the DECT acquisition mode on our system, and the dose-length product (DLP) value was 277.30±35.30 mGy·cm. These values were comparable to the CTDI value of 11.25±3.28 mGy and a DLP value of 265.50±25.90 mGy·cm with a conventional enhanced lung imaging for a patient of normal size at our institution.

### Pathologic Results

Among the total of 63 patients with SPN showing in DECT imaging, 37 (59%) malignant nodules were confirmed, with 19 (30%) adenocarcinoma, 15 (24%) squamous cell carcinoma, and 3 (5%) small cell carcinoma, the residual 26 (41%) nodules were confirmed to be benign. Details are given in Table 1.

**Table 1:** Nodule Diagnosis and Size.

Diagnosis	No. of Nodules (n = 63)	Average Transverse Diameter (mm)
Malignant	37 (59%)	30
Adenocarcinoma	19 (30%)	26
Squamous cell carcinoma	15 (24%)	36
Small cell carcinoma	3 (5%)	28
Benign	26 (41%)	25
Hamartoma	9 (14%)	18
Granuloma	10 (16%)	15
Tuberculoma	5 (8%)	27
Other*	2 (3%)	35

* One sclerosing hemangioma and one chondroma.

### Dual-Energy CT Imaging Characteristics

The four parameters including net CT number enhancement in 70keV images, NIC, WC and λ_HU_ between lung cancers and benign nodules both in AP and VP were compared in Table 2.

**Table 2:** Quantitative Assessment of Net CT number enhancement in 70keV images, Water Concentration (WC), Normalized Iodine Concentration (NIC) and Slope (λ_HU_) between lung cancers and benign nodules in AP and VP.

Group	Net CT numbers (HU)	WC (mg/mL)	NIC	λ_HU_
AP				
Malignant	31.05±28.21	1013.25±58.98	0.41±0.20	2.51±1.20
Benign	2.30±33.72	1006.92±22.08	0.15±0.11	1.31±0.97
*t*	3.919	0.531	8.046	4.549
*P*	<0.001	0.597	<0.001	<0.001
VP				
Malignant	32.47±25.52	1021.52±23.38	0.55±0.23	2.53±1.02
Benign	0.51±31.79	1012.53±21.43	0.23±0.20	1.38±1.06
*t*	4.679	0.949	6.291	4.791
*P*	<0.001	0.345	<0.001	<0.001

After contrast injection, malignant SPNs enhanced more obviously than benign SPNs in AP and VP (Table 2), and statistical differences were shown both in AP (*t* = 3.919, *P*<0.001) and VP (*t* = 4.679, *P*<0.001).

NIC based on material decomposition imaging showed that the iodine concentration in malignant nodules was significantly higher than in benign nodules both in AP (*t* = 8.046, *P*<0.001) and VP (*t* = 6.291, *P*<0.001). Similar results were obtained for λ_HU_ both in AP (*t* = 4.549, *P*<0.001) and VP (*t* = 4.791, *P*<0.001). In contrast, WC in all nodules was statistically the same after enhancement.

For different scan phases, there were significant differences of NIC both for malignant nodules (*t* = -5.996, *P*<0.001) and benign ones (*t* = -3.051, *P* = 0.005). No significant differences of net CT numbers (malignant: *t* = -0.631, *P* = 0.532, benign: *t* = 0.548, *P* = 0.589) and λ_HU_ (malignant: *t* = -0.292, *P* = 0.772, benign: *t* = -0.477, *P* = 0.637) were seen.

### Diagnostic Implication

**Fig 1:**
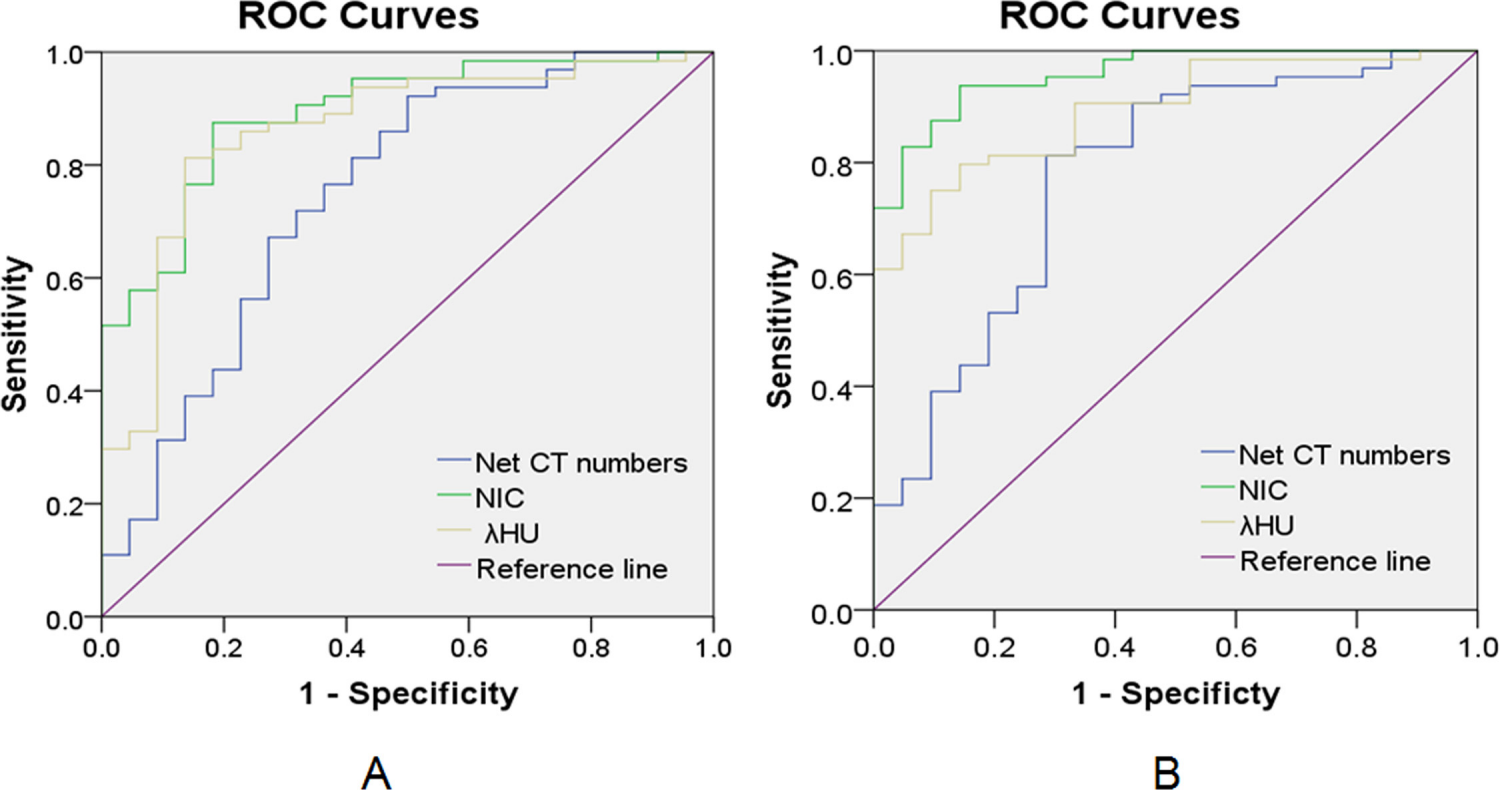
ROC curves of all parameters. ROC curves by using net CT number enhancement, NIC and λ_HU_ to differentiate malignant and benign SPNs in AP (A) and VP (B).

Using ROC curve to further examine the differential capacity with the net CT number enhancement in 70keV images, NIC, WC and λ_HU_ indicated that the net CT number enhancement in 70keV images and WC had poor performances in differentiating malignant and benign SPNs in AP and VP, with the AUC of 0.58–0.66 (Table 3, Fig 1), whereas NIC and λ_HU_ had intermediate to high performances to differentiate benign nodules from malignant ones with the areas under curve of 0.89 and 0.86 respectively in AP and 0.96 and 0.89 respectively in VP. And the sensitivity and specificity of NIC and λ_HU_ were as follows: NIC: 87.5% and 81.8% with threshold of 0.21 in AP, 93.8% and 85.7% with threshold of 0.30 in VP; λ_HU_: 81.3% and 86.4% with threshold of 1.61 in AP, 75.0% and 90.5% with threshold of 1.88 in VP. The diagnostic sensitivity, specificity values are summarized in Table 3.

**Table 3:** Performance of Differential Parameters in Distinguishing malignant and benign SPNs in ROC analysis.

Parameter	AUC	Thresholds	Sensitivity (%)	Specificity (%)
Net CT numbers (AP)	0.65	20	67.7	53.1
Net CT numbers (VP)	0.66	20	74.2	53.8
WC(AP)	0.58	1025.23	56.5	71.9
WC(VP)	0.60	1007.95	87.1	41.9
NIC(AP)	0.89	0.21	87.5	81.8
NIC(VP)	0.96	0.30	93.8	85.7
λ_HU_(AP)	0.86	1.61	81.3	86.4
λ_HU_(VP)	0.89	1.88	75.0	90.5

Using the cutoff of 0.21 in AP and 0.30 in VP for NIC, there were 4 malignant nodules (8.1%, 3/37) having false-negative performances in AP and 1 malignant nodule (2.7%, 1/37) in VP. And for the NIC of the benign nodules, 5 cases (19.2%, 5/26) had false-positive performances in AP and 5 cases (19.2%, 5/26) in VP. With the cutoff of 1.61 in AP and 1.88 in VP for λ_HU_, there were 6 malignant nodules (16.2%, 6/37) having false-negative performances in AP and 6 malignant nodules (16.2%, 6/37) in VP. And for the λ_HU_ of the benign nodules, 5 cases (19.2%, 5/26) had false-positive performances in AP and 4 cases (15.4%, 4/26) in VP.

There were 12 malignant nodules (32.4%, 12/37) which had CT enhancement less than 20HU in AP and 10 nodules (27.3%, 10/37) in VP. Among them, 7 nodules (18.9%, 7/37) had negative enhancement in AP and 2 nodule (5.4%, 2/37) in VP. However, all these nodules except one adenocarcinoma had NIC(AP) >0.21 and NIC(VP) >0.30.

Performances of negative enhancement were also seen in 12 benign nodules (46.2%, 12/26) both in AP and 11 nodules (42.3%, 11/26) in VP. Besides, there were 7 benign nodules (26.9%, 7/26) having CT enhancement more than 20HU both in AP and VP, however, most of them had NIC(AP) lower than 0.21 and NIC(VP) lower than 0.30 except 2 nodules (7.7%, 2/26).

## Discussion

Nowadays, three different hardware approaches to obtain dual-energy data have been proposed and pursued by various vendors: (1) Rapid kVp switching (GE Healthcare, Milwaukee, WI), (2) Energy-sensitive sandwich detectors (Philips Medical Systems, Cleveland, OH) and (3) Dual-source, dual-energy CT (Siemens Healthcare, Forchheim, Germany) [20]. In rapid kVp switching the x-ray tube rapidly modulates the tube voltage, thus simultaneously producing lower and higher energy spectra. This switching procedure requires doubling the number of projections per rotation to maintain sufficient data for image reconstruction [20]. Energy-sensitive sandwich detector technology uses a layer detector design. Both detectors are superimposed on each other and are composed of different materials. The top-layer detector absorbs low energy x-ray photons, whereas the bottom-layer detector detects higher-energy x-ray photons. Similar to rapid kVp switching, the low and high tube voltage output is limited to the same filtration [20]. For the dual-source, dual-energy CT, two tubes with different kVp (80 and 140kVp) and two corresponding detectors are used [21]. It can significantly reduce the spectral overlap of the low-energy spectrum with the high-energy spectrum [21]. The system in our study was rapid kVp switching.

In this study we investigated the potential of using quantitative information provided by both the monochromatic images and material decomposition images in dual-energy spectral CT imaging for the differentiation of benign and malignant SPN. The statistical analysis showed significant differences between benign and malignant SPN in NIC, λ_HU_ and net CT numbers enhancement in 70keV images (both in AP and VP, all *P*<0.001). These results suggested that iodine content after enhancement in nodules could be used as a quantitative parameter to distinguish malignant nodules from benign ones.

In previous studies for SPNs, net CT number increases between plain and contrast enhanced scans were often used for differentiating benignity and malignancy, and 20 or 15HU has been suggested as a cutoff value [8–10,16]. Due to the high vascularity in tumors and interstitial accumulation of contrast material by means of increased permeability of tumor capillaries [15], tumors would performed rapid and strong contrast enhancement. In our study, the net CT number increases of malignant SPNs in the 70keV images after contrast enhancement were significantly higher than those of benign SPNs, indicating stronger enhancement in tumors. This is consistent with the previous studies [9,10,16]. However, our study also indicated that the potential for diagnostic differentiation of benign and malignant SPNs with the net CT number change after the administration of contrast materials in 70keV images was limited. Using the threshold of 20HU for CT number change in VP, we obtained sensitivity of 74.2% and specificity of 53.8% for differentiating malignant from benign SPN. The sensitivity value was similar to but the specificity value was slightly lower than the corresponding value of 72% and 71.1% reported in a previous study with the same cutoff value [16]. The use of CT number difference to separate malignant and benign tumors is limited by many factors in imaging, including the size of tumors, the impact of beam hardening effects as well as the average photon energy of the x-ray source used. In addition, there may be energy difference between the 70keV for the enhanced images and the 120kVp for the nonenhanced images in our study, introducing possible systematic errors for the CT number difference measurement. On the other hand, quantitative parameters, such as the normalized iodine concentration and slope of spectral HU curve that are specific to the dual energy spectral CT imaging, provided much greater ability to separate malignant and benign tumors. Our study indicated that by using a threshold value of 0.30 for the normalized iodine concentration in the venous phase one could obtain a sensitivity of 93.8% and specificity of 85.7% for differentiating malignant from benign SPN in lungs. These values were statistically higher than those with the use of CT enhancement.

There were several nodules (7 cancerous nodules (18.9%, 7/37) in AP and 2 (5.4%, 2/37) in VP, and 12 benign nodules (46.2%, 12/26) in AP and 11 (42.3%, 11/26) in VP) which had negative enhancement values. This phenomenon has been observed in earlier studies and could be related to intrascanner variation, sampling differences in the region of interest of an inhomogeneous nodule or beam-hardening artifact [22]. Besides, the CT enhancement in our study were a bit lower than the previous researches [9,10]. This phenomenon was possibly due to sampling differences in the region of interest of an inhomogeneous nodule, or possible systematic errors for the CT number difference measurement due to the energy difference between the 70keV for the enhanced images and the 120kVp for the nonenhanced images in our study. However, a DECT could simultaneously provide the iodine-based material decomposition images and a set of monochromatic images at energies ranging from 40 to 140keV from a single scanning; therefore, we can acquire the value of normalized iodine concentration and slope of a spectral curve from the same ROI. This technique may reduce measurement error due to different positioning of the ROI on sequential images. Effect on measurement error might be more important in the relatively smaller nodules [16].

Angiogenesis is a fundamental process in the development of tumors, whereby the growing malignancy appropriates its own blood supply from adjacent tissues. The results of stronger enhancement of malignant tumors on CT [9,10,16] or MRI [15] are based on the richer angiogenesis of malignant tumors than benign nodules. Iodine, being the main ingredient of contrast medium, directly reflects the blood flow and distribution in the intravascular and extracellular spaces, and iodine concentration maps are often regarded as having potential for assessing the relative vascularity and vasculature of pulmonary nodules [16,17]. Several studies have proved that iodine concentration on iodine images were significantly different between inflammatory and malignant lung masses [6,23]. However, the differences of iodine concentration between benign and malignant tumors have not been evaluated using DECT so far. And as far as we know, we were the first to make quantitative analysis of the difference between benign tumor and lung cancer using DECT. Our results shown that the iodine concentration in malignant nodules was significantly higher than benign nodules both in AP (*P*<0.001) and VP (*P*<0.001), and could be used to provide high accuracy to differentiate the two types of lesions.

In general, the combination of photo-electric and Compton effects contributes to CT attenuation of any material, and these two mechanisms are both energy- and material-dependent. By analyzing the two x-ray spectra acquired simultaneously, dual-energy imaging allows the analysis of Compton and photoelectric effects to express any material using a basis material pairs, and to form virtual monochromatic image sets at single energy levels. Therefore, in a polychromatic examination, different monochromatic datasets could be acquired, which provide information about the attenuation changes in different materials as a function of x-ray photon energy. This approach can be effective in tissue characterization through analysis of attenuation curves of different materials at distinct energy levels [17,20]. This is different from the conventional CT where only CT number at the average photon energy is available.

**Fig 2:**
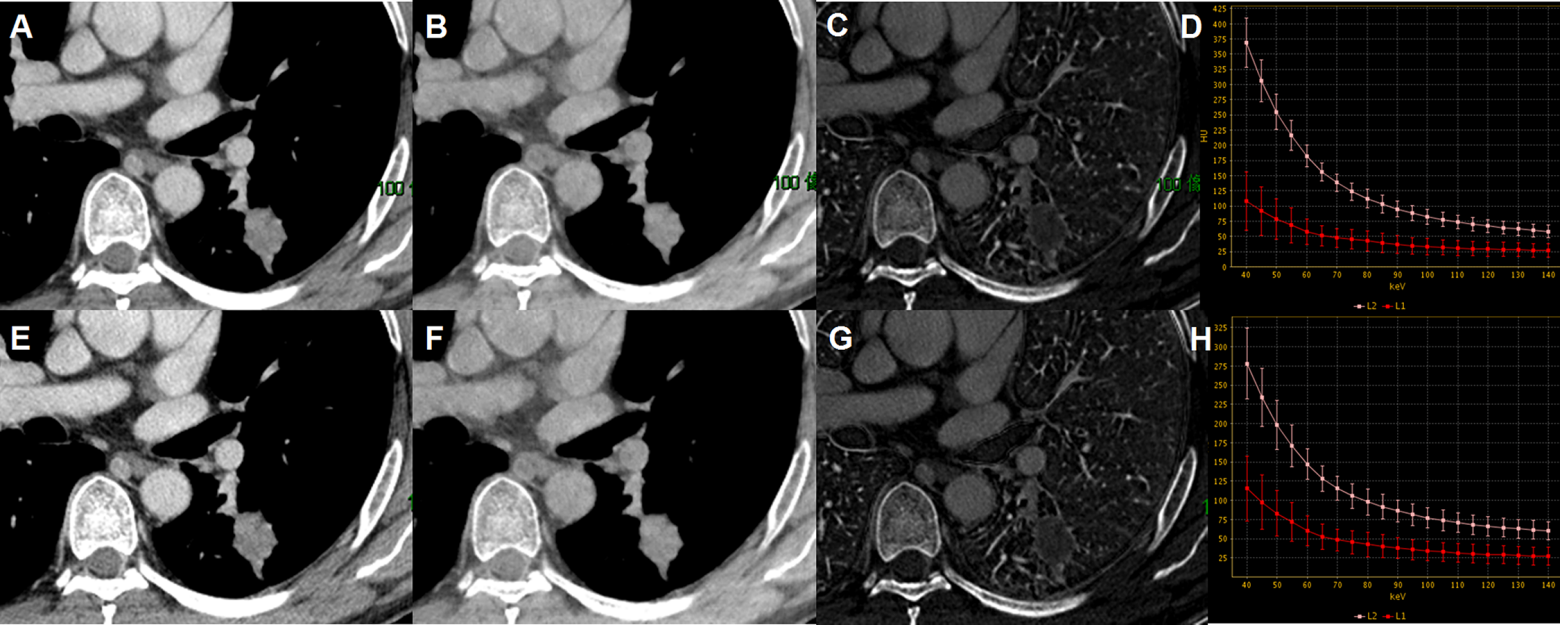
Spectral CT images of adenocarcinoma. Spectral CT images of a 46-year-old man with adenocarcinoma in AP (A-D) and VP (E-H): 70 keV images after enhancement (A, E), Water-based images (B, F), Iodine-based images (C, G) and spectral curves (D, H). The spectral HU curves were respectively arterial (L2, the pink curve) and tumorous lesion (L1, the red curve) (D, H). Net CT numbers (AP) = 19.65, Net CT numbers (VP) = 14.23, NIC(AP) = 0.29, NIC(VP) = 0.38, λ_HU_(AP) = 1.45, λ_HU_(VP) = 1.39.

**Fig 3:**
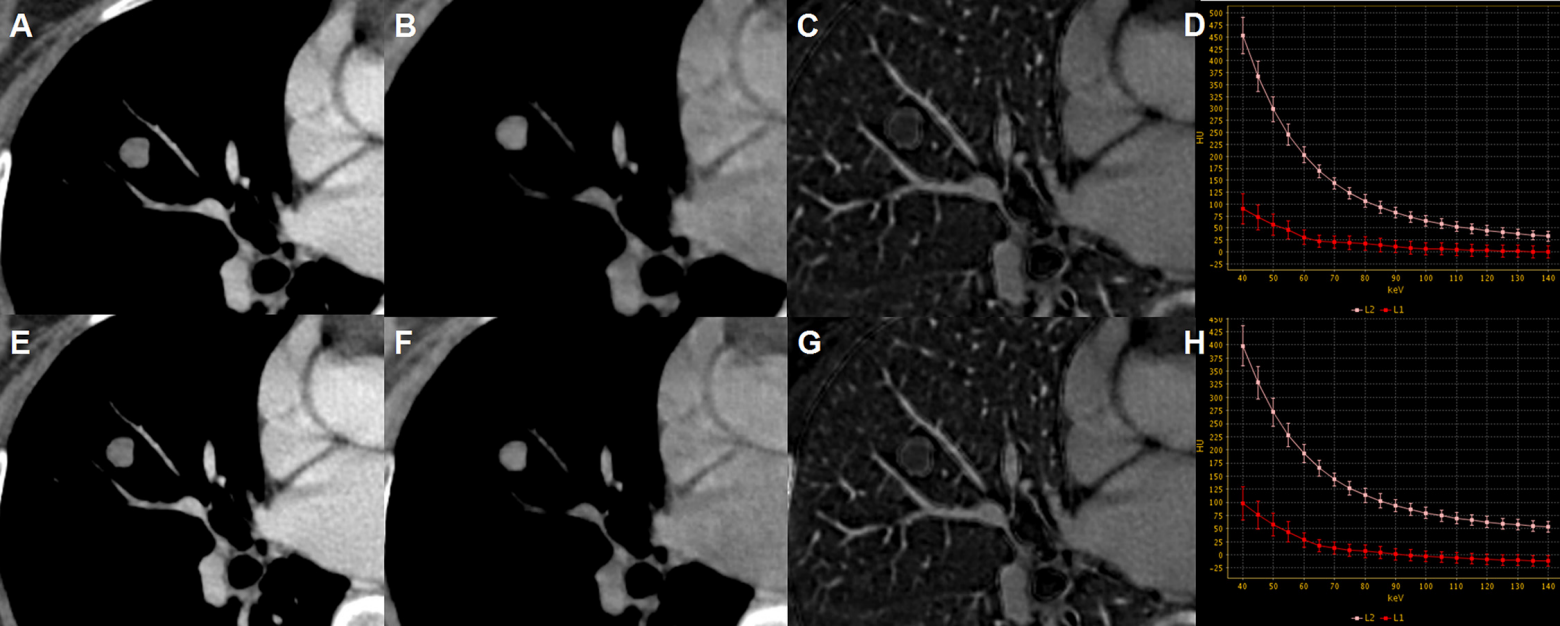
Spectral CT images of hamartoma. Spectral CT images of a 50-year-old woman with hamartoma in AP (A-D) and VP (E-H): 70 keV images after enhancement (A, E), Water-based images (B, F), Iodine-based images (C, G) and spectral curves (D, H). The spectral HU curves were respectively arterial (L2, the pink curve) and tumorous lesion (L1, the red curve) (D, H). Net CT numbers (AP) = -0.35, Net CT numbers (VP) = -15.64, NIC(AP) = 0.10, NIC(VP) = 0.18, λ_HU_(AP) = 1.06, λ_HU_(VP) = 1.30.

With GSI viewer software, attenuation curves or HU curves could be automatically generated for given ROIs, describing the dynamic change of measured CT Hounsfield units of ROIs against photon energy values within the range of 40 to 140keV (Figs 2 and 3). To facilitate the description of HU curve, a nominal parameter, the slope of HU curve (λ_HU_) was established. A previous study [23] had shown that λ_HU_ between inflammatory and malignant masses had significant difference. This is owing to the greater attenuation of iodine at lower energies [24]. And this phenomenon further indicated that the enhancement difference could be amplified between the two types of pulmonary nodules when low photon energy was used.

Surely, the lower the energy, the better SPNs are distinguishable because of the spread in the curves. However, the low energy also significantly increases noise, and is patient size-dependent. The benefit of using lower energy to balance contrast and image noise to achieve optimal signal-to-noise ratio needs further investigations.

Our study had several limitations. First, the evidence in the subgroups was limited by the relatively small study population, especially benign tumors. Further study with more cases needs to be performed in the future. Second, the diameters of masses in our study were relatively large. However, the results also showed that both NIC and λ_HU_ obtained from spectral CT imaging could improve the differentiation between malignant and benign SPNs in comparison with CT enhancement in a quantitative way. We are not sure whether there is any correlation between NIC or λ_HU_ and diameters of the SPNs. And further investigations are needed, the same with additional studies of SPNs with diameter less than 30mm, especially around 10mm. Third, although we had compared the positions and sizes of ROIs between different sequences carefully, there were still possible sampling differences for different sequences in our study, especially for the calculations of CT numbers between nonenhanced and enhanced images. Thus net CT enhancement values might be influenced by these errors. However, a DECT could simultaneously provide the iodine-based material decomposition images and a set of monochromatic images at energies ranging from 40 to 140keV from a single scanning; therefore, we can acquire the value of normal iodine concentration and slope of a spectral curve from the same ROI. This technique may reduce measurement error due to different positioning of the ROI on sequential images. Forth, the differentiation of inflammatory nodules and lung cancer are quite difficult sometimes. However, inflammatory nodules were not included in this study. There are some previous studies which had made efforts to find out whether spectral CT imaging are useful for the differentiation of the malignant and benign SPNs [6,23]. And the results showed that spectral CT imaging were helpful for the differentiation of the malignant and benign SPNs. The limitation of small number of cases exited in these researches (with 24 cases of pneumonia and 44 cases of lung cancer [6], 25 cases of inflammatory masses and 35 cases of lung cancer [23], respectively). And further studies with more cases are needed. Fifth, specific morphologic features are useful in determining the malignant potential of a nodule [3], and these were not included in our study. We believe that the combinations of morphological and quantitative evaluations of SPN might improve the diagnostic accuracy, and further study is needed. Additionally, we did not analyze calcification in nodules, which might be a useful imaging feature to distinguish benign SPNs from malignant SPNs [2,25]. However, there are about 45% of benign nodules being calcified [26], thus, other imaging features associated with benignity must be sought.

## Conclusions

DECT imaging provides promising quantitative approach for distinguishing malignant SPNs from benign ones. And the iodine content and slope of HU curve obtained in dual energy spectral CT could be valuable parameters for the differentiation of SPNs.

## Supporting Information

S1 Dataset Data of our study. (SAV)
